# Innate humoral immune defences in mammals and insects: The same, with differences ?

**DOI:** 10.1080/21505594.2018.1526531

**Published:** 2018-10-13

**Authors:** Gerard Sheehan, Amy Garvey, Michael Croke, Kevin Kavanagh

**Affiliations:** Department of Biology, Maynooth University, Maynooth, Ireland

**Keywords:** Antimicrobial peptide, coagulation, humoral immunity, insect, mammal, melanization, Toll

## Abstract

The insect immune response demonstrates many similarities to the innate immune response of mammals and a wide range of insects is now employed to assess the virulence of pathogens and produce results comparable to those obtained using mammals. Many of the humoral responses in insects and mammals are similar (e.g. insect transglutaminases and human clotting factor XIIIa) however a number show distinct differences. For example in mammals, melanization plays a role in protection from solar radiation and in skin and hair pigmentation. In contrast, insect melanization acts as a defence mechanism in which the proPO system is activated upon pathogen invasion. Human and insect antimicrobial peptides share distinct structural and functional similarities, insects produce the majority of their AMPs from the fat body while mammals rely on production locally at the site of infection by epithelial/mucosal cells. Understanding the structure and function of the insect immune system and the similarities with the innate immune response of mammals will increase the attractiveness of using insects as *in vivo* models for studying host – pathogen interactions.

## Introduction

The innate immune system is the first line of defence against invading pathogens in both insects and mammals. Although innate immune responses are non-specific, they are widely distributed throughout the body allowing them to play a crucial role in the maintenance of homeostasis and the prevention of disease and infection [1]. The insect and mammalian innate immune systems consist of humoral and cellular responses. The cellular response is mediated by hemocytes in insects and myeloid cells in mammals and involves the targeting of pathogens through processes such as phagocytosis, superoxide production, encapsulation and enzyme release. Insect hemocytes display many structural and functional similarities to neutrophils of the mammalian immune response [2–4]. Insect and mammalian humoral responses involve processes such as melanization, clotting and the secretion of antimicrobial peptides. In addition, mammals also possess an adaptive immune system which first evolved in jawed fish 500 million years ago after the divergence of vertebrates and invertebrates [5]. The adaptive immune system enables a specific response to a pathogen and relies upon the presence of lymphocytes with specific receptors that recognize pathogenic antigens. The adaptive immune system also has the ability to remember previous pathogen attacks, resulting in a more effective immune response to subsequent infection [6]. While insects do not have an adaptive immune response they display immunological priming as a result of prior exposure which enhances survival to a subsequent insult as a result of increased humoral and cellular responses [4,7,8].

A wide range of insects is now employed to study the virulence of medically important pathogens and this is made possible by the similarities between the insect immune response and the mammalian innate immune responses. Insects such as *Drosophila melanogaster* [9–11], *Galleria mellonella* [12–15], *Manduca sexta* [16,17], and *Bombyx mori* [18] are now widely used to overcome the disadvantages associated with testing in mammalian systems (e.g. cost, housing, legal/ethical restrictions) while generating comparable results. This development has accelerated research and lead to a reduction in cost and in the use of mammals for these types of experiments [19–22]. This review compares the humoral immune response of insects and mammals and demonstrates how these can be exploited to validate the use of insects as alternatives to the use of mammals.

## Humoral immune signalling pathways in mammals and insects

### The Toll and Toll-like pathway

Toll-like receptors (TLRs) are a group of type I transmembrane receptors that play a role in innate humoral immunity in both insects and mammals. As Toll and TLRs are conserved throughout evolution, they can be found in mammals, invertebrates and plants. Homologies between these receptors can be observed between the cytoplasmic Toll/IL-1R (TIR) domain of both mammalian Toll-like receptors and the *Drosophilia* Toll receptor. In addition to a TIR cytoplasmic domain, toll receptors can be characterized by an extracellular domain consisting of several leucine-rich repeats (LRRs) [23]. Despite the conservation of these domains in mammals and insects there are some structural and functional differences observed in the individual Toll receptors and genes. Structurally, mammalian Toll-like receptors contain one cysteine cluster at the C-terminal domain of their LRRs while insects contain multiple cysteine clusters on both the C-terminal and N-terminal domains of their LRRs [24]. Functionally, the Toll genes are vital in *Drosophilia* embryogenesis where they are involved in dorsal-ventral development. Insect Toll-signalling is also essential in the production of AMPs in response to pathogen invasion, in particular antifungal peptides such as drosomycin, highlighting its role in innate humoral immunity [25]. Conversely, mammalian Toll-like genes are involved in the production of cytokines and co-stimulatory molecules upon pathogen recognition. The production of these molecules results in the activation of T-lymphocytes, thereby linking the mammalian innate and adaptive immune responses [26].

**Figure 1.**
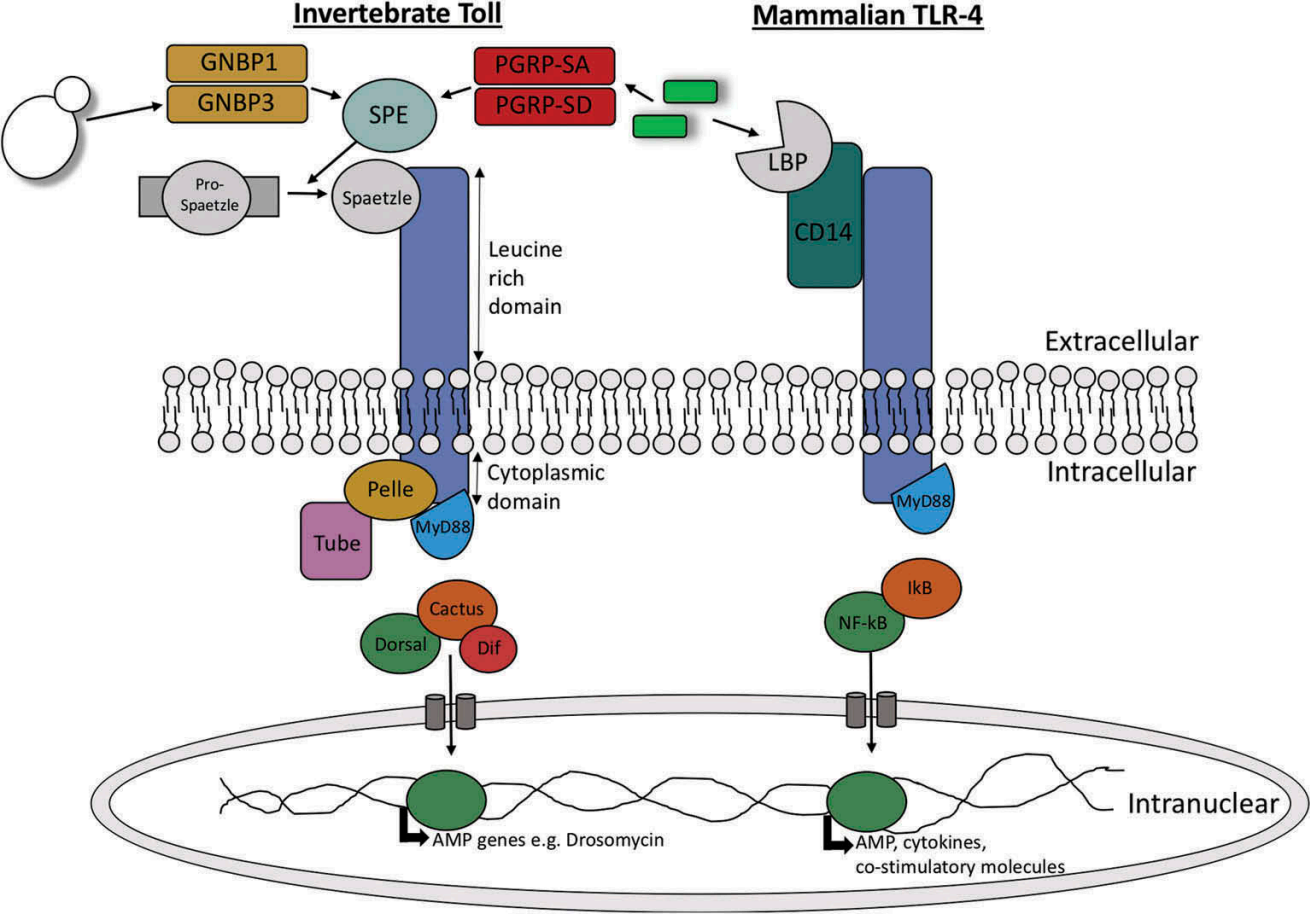
Diagramatic representation of the similarities between invertebrate Toll signalling and vertebrate toll-like signalling. Upon activation of invertebrate toll receptor and the homologous toll-like receptor in vertebrates, a cascade is induced where the homologous transciption facotors Nf-κB and Dif are activated in vertebrates and invertebrates, respectively. Upon translocation of these transcription factors, AMPs are produced in invertebrates while co-stimulatory molecules and pro-inflammatory cytokines such as IL-1β, IL-6 and IL-8 are produced in vertebrates.

Upon activation of Toll and Toll-like receptors a series of similar signalling pathways is initiated in insects and mammals which lead to activation of homologous transcription factors; NF-κB in mammals and Dorsal and Dif in insects (Figure 1). These transcription factors are responsible for the resulting anti-microbial response in both animal groups [27]. Toll pathway activation in mammals occurs directly through binding of microbial associated material to their specific Toll receptor. In insects, activation occurs indirectly where microbial invasion induces the production of a cysteine-knot protein called Spätzle which can bind to Toll receptors [28]. Upon *Drosophilia* Toll activation by Spätzle the adaptor protein myeloid differentiation primary response protein (MyD88) is recruited to the TIR domain of the Toll receptor. A hetero-trimeric complex is formed with MyD88, the kinase Pelle and the protein Tube. As a result of Pelle activation, the inhibitor protein Cactus is phosphorylated and degraded. In its inactive form Cactus is bound to the transcription factors Dif or Dorsal. Therefore, upon Cactus degradation Dorsal or Dif are free to translocate to the nucleus resulting in the transcription of anti-fungal AMPs [29]. A very similar proccess occurs in mammalian Toll-like signalling (Figure 1). In mammals, upon binding of microbial derived material such as peptidoglycan or lipopolysaccharide to Toll-like receptor MyD88, which is homologous to *Drosophila* MyD88, is recruited to the TIR domain. This initiates the recruitment of IRAK kinases which are homologous to *Drosophila* Pelle and Tube. Kinase activation results in the phosphorylation and degradation of I-κβ, which is homologous to *Drosophila* Cactus, thereby initiating the translocation of the now unbound NF-κB (homologous to *Drosophila* Dif and Dorsal) to the nucleus for transcription of co-stimulatory molecules, cytokines and chemokines [30].

### The IMD and TNF-α signalling pathways

**Figure 2.**
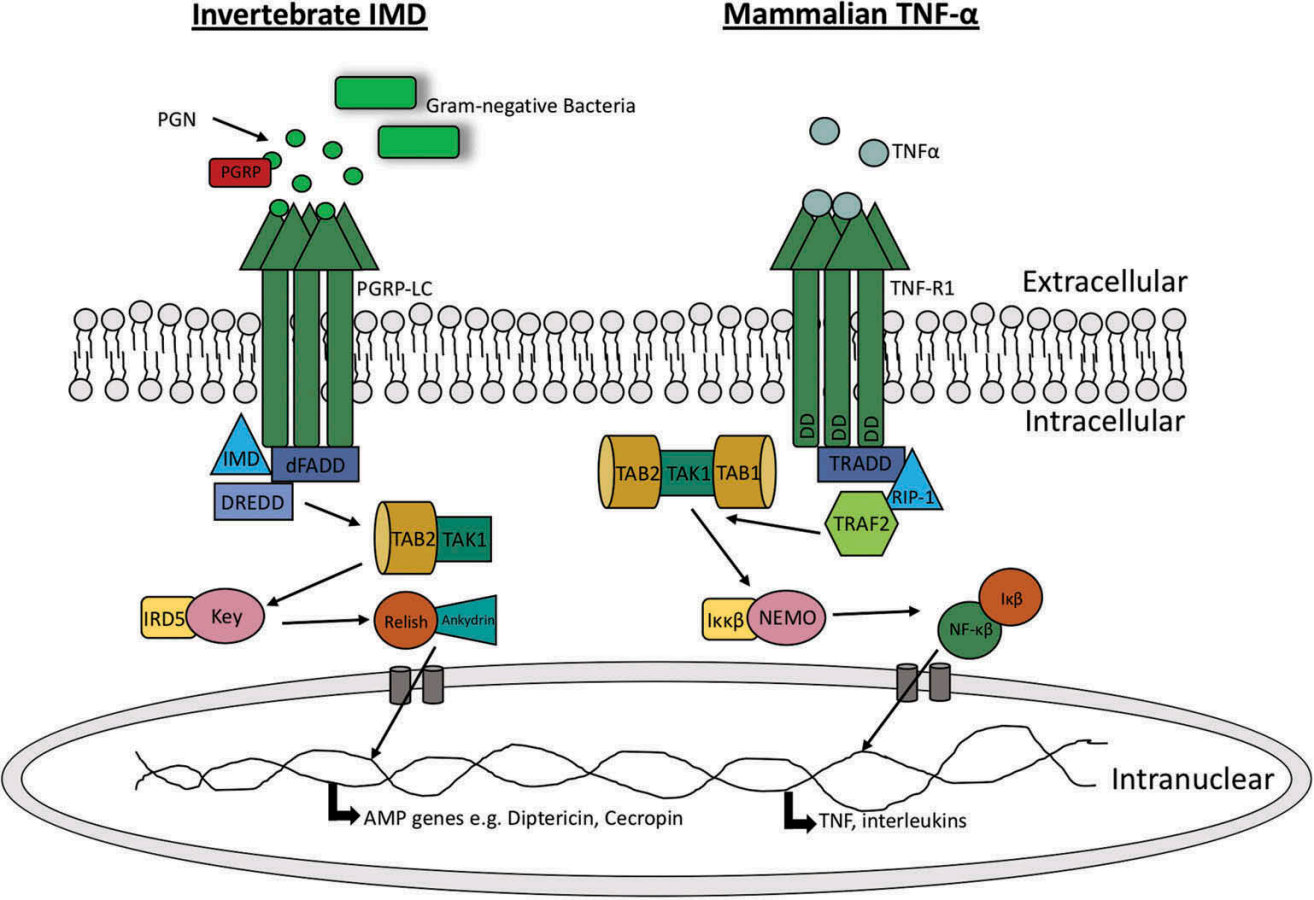
Comparision of insect IMD pathway and mammalian TNF-α pathway. The IMD pathway is activated by binding of peptidoglycan (PGN) to peptidoglycan-recognition proteins (PGRPs) which results in recruitment and formation of a IMD, dFADD and DREDD complex and results in IMD cleavage and subseqeunt activation of TAB2/TAK1. This results in Relish phosphorylation and ultimately the production of AMPs (e.g cecropin). Alternatively in mammals, TNF-α is bound by the tumor necrosis factor receptor 1 (TNF-R1) which results in recuirment of RIPP, FADD and caspase 8. This complex activates TAK1 which activates the IKK complex resulting in phosphoylation and degradation of the inhibitor protein IκB. NF-κB is released for translocation to the nucleus resulting in pro-inflammatory cytokine production.

In addition to Toll signalling, *Drosophilia* can induce the IMD signalling pathway to produce AMPs. The IMD pathway recognizes components of the bacterial cell wall such as peptidoglycan, resulting in the activation of a cascade that ultimately produces AMPs. The IMD pathway displays some similiarities to the mammalian tumour necrosis factor-α (TNF-α) pathway as well as the Toll-like pathway. Both the TNF-α and IMD pathways ultimately result in the production of the homologous transcription factors NF-κB and Relish, respectively (Figure 2). In insects, the IMD pathway is initiated by binding of peptidoglycan to peptidoglycan-recognition proteins (PGRPs) while in mammals, the TNF-α pathway is initiated through the binding of TNF-α to tumor necrosis factor receptor 1 (TNFR1) [31,32]. Peptidoglycan recognition in *Drosophila* results in the recruitment of IMD, a death domain protein, the adaptor protein dFADD, and DREDD to form a complex. The activation of this complex promotes the clevage of IMD from the complex and activation of the *Drosophila* TAB2/TAK1 complex. As a result, the *Drosophila* IKK complex is activated and phosphorylates Relish. Rel-68, the N-terminal domain of Relish, can then translocate to the nucleus and initiate the production of anti-bacterial AMPs such as diptericin [33]. Conversely, TNFR1 activation in mammals initiates the recruitment of RIPP, FADD and caspase 8 which are homologous to *Drosophila* IMD, dFADD and DREDD, respectively. The formation of the RIPP/FADD/caspase 8 complex activates TAK1 (homologous to *Drosophila* TAK1). TAK1 activates the IKK complex (homologous to *Drosophila* IKK complex) which phosphoylates and degrades the inhibitor protein IκB. Upon IκB degradation, NF-κB is released for translocation to the nucleus [34]. In mammals, TNF pathways are involved in the production of pro-inflammatory cytokines as well as cell survival and apoptosis pathways, therefore also linking the innate and adaptive immune responses [35].

**Table 1.** A comparison of the antimicrobial peptides present in insects and humans.

*Drosophila melanogaster*		*Galleria mellonella*		Human	
**Name**	**Characteristic**	**Name**	**Characteristic**	**Name**	**Characteristic**
**Cecropins**	α helical	**Cecropins**	α helical	**Cathelicidin**	α helical
**Drosomycin**	Cysteine rich	**Gallerimycin**	Cysteine rich	**Defensin**	Cysteine rich
**Metchnikowin**	Proline rich	**Galliomicin**	Cysteine rich	**Histatin-5**	Histidine rich
**Attacins**	Glycine rich	**Moricin-like peptides**	α helical	**Dermcidin**	Anionic peptide
**Drosocin**	Proline rich	**Gloverin-like peptides**	Glycine rich

**Table 2.** A comparison of humoral receptors, anti-microbial peptides, cascades and enzymes in mammalian and insect humoral immune responses.

	**INSECT**	**MAMMALIAN**
**RECEPTORS**	Toll, IMD, β-1,3 glucan, IL-1R, Calreticulin, Hemolin, Lectins, Hemocytin	TLRs, TNFα, β-1,3 glucan, IL-1R, Calreticulin, C-type lectins, Macrophage mannose receptor
**TRANSCRIPTION FACTORS**	NF-κβ, I-κβ	NF-κβ, I-κβ
**COAGULATION CASCADE**	Transglutaminase, Hemolectin	Factor XIIIa, vWF
**IMMUNE CASCADES**	Prophenoxidase cascade	Complement cascade, Melanization of skin
**METALLOPROTEINASE INHIBITORS**	IMPI	Collagenase, Gelatinase
**AM-PEPTIDES**	Defensins, cecropins, moricins, gloverins, attacins	Defensins, LL-37, Dermcidin
**AM-PROTEINS**	Lysozyme, Sarcotoxin	Lysozyme, Histatin-5

## The blood/hemolymph clotting system in insects and mammals

### The role of clotting systems

Clotting is an essential component of the innate immune system and promotes hemostasis by inducing the formation of an insoluble matrix/clot in the insect hemolymph or mammalian blood. The formation of a clot also aids in the sealing of wounds and prevention of pathogen entry and infection [36]. Due to insects having an open circulatory system, clotting plays a vital role in the insect immune system. The insect clotting system is extremely efficient in order to prevent loss of hemolymph and the spread of infection, and for hemocoel compartmentalisation. Additionally, clotting helps to limit potential tissue damage caused by other immune responses by localising activity to wounding/pathogen entry sites. In contrast, mammals have a closed circulatory system and an adaptive immune response which lessens their reliance on such an efficient clotting system, especially since thrombosis (clot formation) is costly [37].

### Similarities between insect and mammalian clotting factors

**Figure 3.**
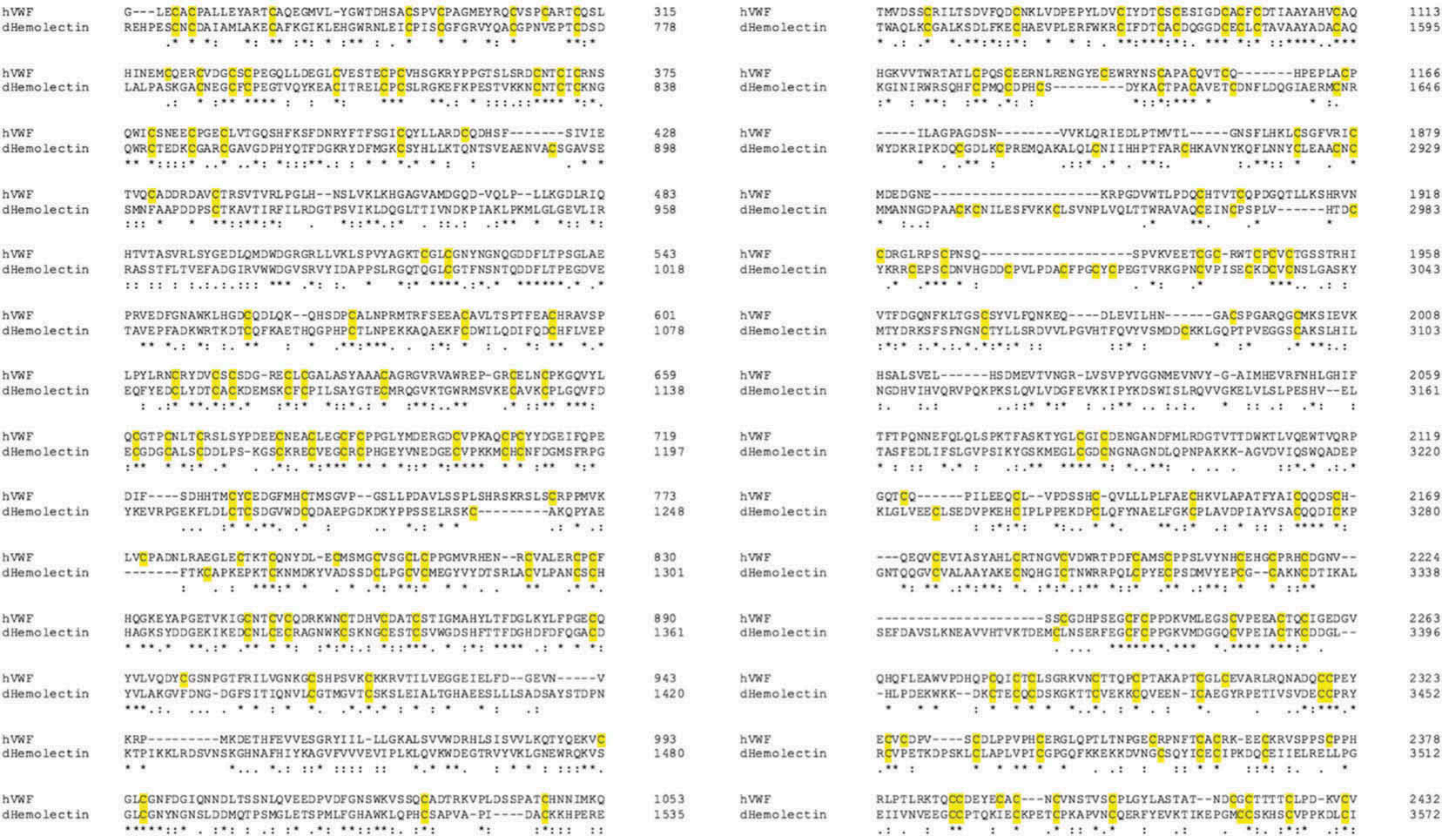
Sequence comparison between human (h) von Willebrand factor and *Drosophila* (d) Hemolectin protein sequences using emboss needle pairwise sequence alignment. Highlighted in yellow are conserved cysteine residuals. hVWF and dHemolectin are up to 30.9% similar protein sequences. * (asterics); indicates a single, fully conserved residue,: (colon); indicates conservation between groups of strongly similar properties, . (full stop); denotes conservation between groups of weak similar properties).

Similarities between insect and mammalian clotting cascades can be observed in the family of transglutaminases which are involved in the hardening of a clot. Insect transglutaminases are homologous to human clotting factor XIIIa; one of eight transglutaminases found in humans. Factor XIIIa is involved in the final hardening of the clot in humans while insect transglutaminase is believed to contribute to the clotting cascade at a much earlier stage [38,39]. Additionally, homologies have been observed between domains of the *Drosophila* clot fibers constituent hemolectin and domains of human clotting factors V and XIII. Similarities in protein sequence have also been observed between insect hemolectin and human von Willebrand factor (vWF), a glycoprotein involved in hemostasis, (Figure 3) [40,41].

**Figure 4.**
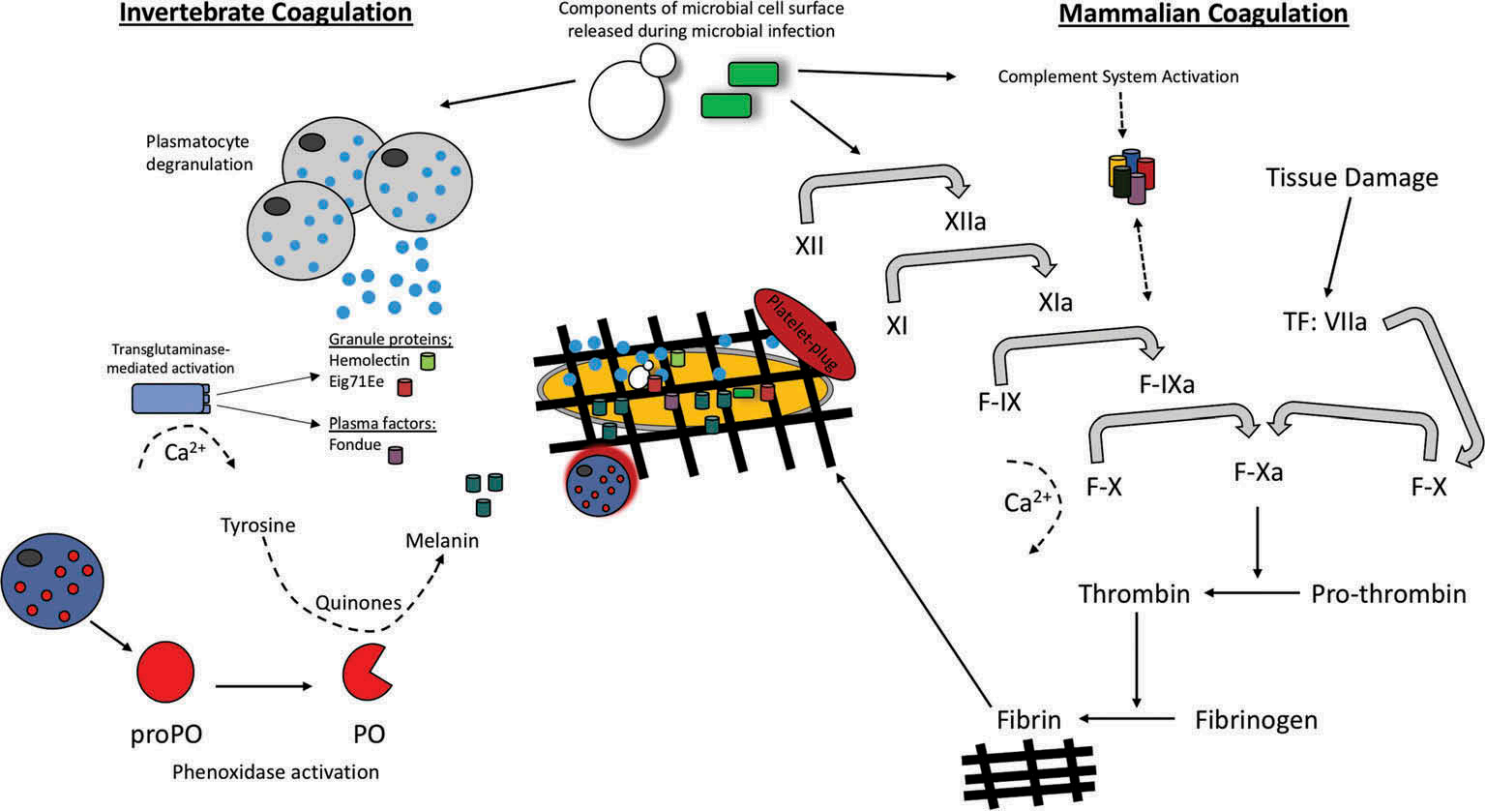
Schematic comparison of the hemolymph/blood clotting system in insects versus mammals. Hemolymph clotting in insects involves co-ordination between plasmatocytes, transgluatimase mediated activation of hemolectin, eig71Ee and fondue as well as phenoxidase activation. During the mammalian blood clotting a series of enzymatic reactions result in the formation of thrombin and subsequently the conversion of fibrinogen to fibrin. Activations of the complement cascade also feeds into mammalian coagulation in the same way as phenoloxidase activation in insects. Similarities can be seen in both systems in that TG is homologous to factor XIIIa. Both factors contribute to the formation of a hemolymph/fibrin network.

Despite the conservation of structure and functions of some clotting factors, greater variation in clotting cascades and clotting factors has been observed between insects and mammals. *Drosophlia* clotting cascades involve a number of clotting factors in three clotting steps to form a hardened clot. Firstly, a primary clot is formed through hemocyte degranulation where an aggregate is formed consisting of hemocytes, cell debris and extracellular matrix [42,43]. Secondly, the prophenoloxidase system (see: “The insect prophenoloxidase activating (proPO) system”) and transglutaminases are activated which contribute to the crosslinking and hardening of the clot [44]. This also highlights the link in evolution of melanization and clotting in insects, a process that occurs independently in mammals. Finally, plasmatocytes are recruited to seal the clot [45]. In *Drosophila*, a number of clotting factors have been identified. Hemolectin is the most abundant protein found in the insect clot but many other factors contribute to the formation of a stable clot [46]. Activated transglutaminase (as a result of wounding or infection) interacts with its substrates Fondue and Eig71Ee, causing their subsequent covalent cross linkage and the formation of a hardened clot, (Figure 4) [47]. Lipophorin, which is analogous to mammalian lipid carrrier, is also involved in the polymerisation of the insect clot. Additionally, the activation of phenoloxidase by the proPO system works alongside transglutaminase through its involvement in the final crosslinking of the clot in addition to having a primary function in the direct killing of pathogens through melanization [46].

Mammalian clotting systems are better understood and more clotting factors have been identified. Firstly, primary hemostasis in humans occurs where a platelet plug in formed at the site of bleeding. Secondly two different pathways can be activated both of which lead to fibrinogen being converted to fibrin; the extrinsic tissue factor (TF) system or the intrinsic contact system. The contact system involves activation of factor XII by collagen exposure in the bleeding vessel. Subsequently, the activation of the factors XI, IX, VIII and X takes place, respectively. Factor X (in the presence of lipids, platelets, calcium and factor V) initates the conversion of prothrombin to thrombin which then converts fibrinogen to fibrin. The production of fibrin finally stabilizes the fibrin network/clot [48,49]. TF is constitutively expressed smooth muscle cells, pericytes and fibroblasts. Upon vessel injury platelets bind to vWF. This event then initiates the binding of TF to factor VIIa resulting in platelet activation via PAR1 and PAR4 receptors. Platelet activation ultimately leads to the conversion of prothrombin to thrombin and finally fibrinogen to fibrin, as in the contact system (Figure 4) [50,51].

## Melanization in insects and mammals

Melanins are a group of pigmented biopolymers dervived from phenolic compounds such as tyrosine. Melanins are believed to have evolved over 500 million years ago and can be found in both insects and mammals despite having a different primary role in both groups. In mammals, the production of melanin pigments is an important component in the colouration of hair, eyes and skin but also contributes to protection from solar radiation [52]. In insects, melanin production plays a vital role in the innate immune system, colouration of the exoskeleton, sclerotization and healing of wounds. There are three types of melanin in mammals; pheomelanin which are the yellow-red pigments found in red hair/fur/feathers, eumelanin which are the black to brown pigments and neuromelanin which consists of polymeric components derived from dopamine and produced by the substantia niagra in the brain [53,54].

### The insect prophenoloxidase activating (proPO) system

In insects melanin production depends upon the activation of the prophenoloxidase activating (proPO) system which is rapidly triggered upon pathogen invasion or injury to the cuticle. Upon activation of the proPO system, a pathogen can be killed directly through the production of toxic compounds. Alternatively, phagocytosis and encapsulation of the invading pathogen or hemolymph coagulation may also be induced by this system [55]. The proPO system cascade leading to the production of melanin is catalysed by the redox enzyme phenoloxidase. Mammalian melanin production is also catalysed by a redox enzyme called tyrosinase and this has similar activity to insect phenoloxidase although they share little homology. These enzymes also differ in where they are located; tyrosinase is membrane-bound within the melanosome of mammals while phenoloxidase is produced by the insect hemocytes and secreted into the hemolymph upon activation [56].

**Figure 5.**
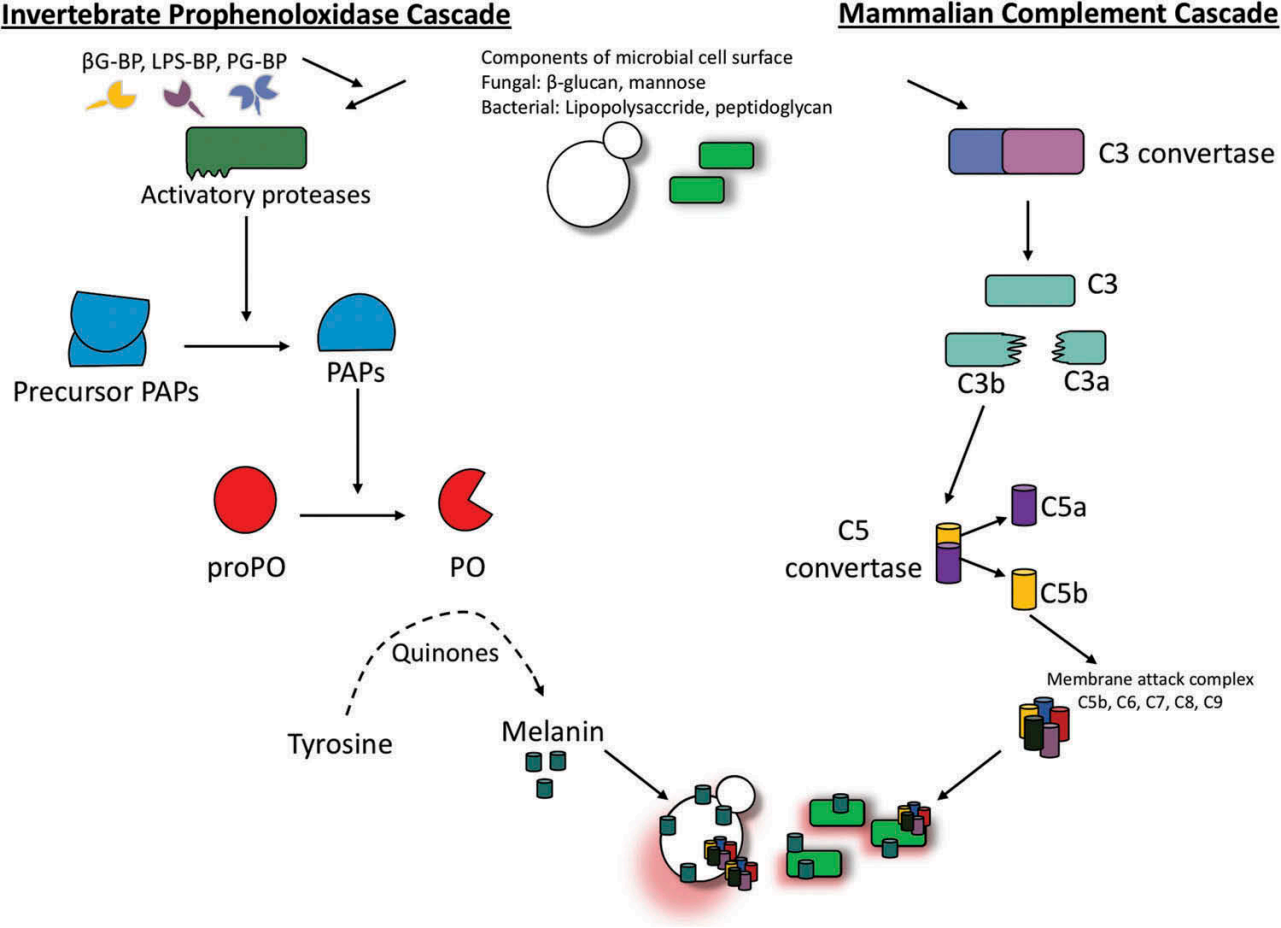
Schematic diagram of the proPO-system for melanin production in insects. PAMPs such as β-1,3 glucan, LPS and peptidoglycan amongst others bind pathogen recognition receptors such as β-1,3 glucan-binding protein (βG-bp), lipopolysaccharide-binding protein (LPD-BP) and peptidoglycan-binding protein (PG-BP), respectively. This results in the activation of the serine protease cascade which initates the conversion of prophenoloxidase-activating enzyme from its pro-form (pro-ppA) to its active form (ppA). PpA then catalyzes the conversion of prophenoloxidase (proPO) to phenoloxidase (PO). PO in combination with phenols and O_2_ results in the formation of quinones which polymerize to form melanin. Similarily, the alternative complement pathway generates C3b by C3 convertase which with other proteins froms the C5 convertage. This enzyme cleaves C5 to C5a and C5b, the latter of which recuits and assembles C6, C7, C8 and multiple C9 molecules to form a pore forming membrane attack complex which is deposited on the microbial cell surface ultimately resulting in cell lysis.

Melanization in insects involves a number of cascades that must be carefully regulated due to the production of toxic and reactive intermediates which may be detrimental to the host. ProPO activation can be triggered by pathogen associated molecular patterns (PAMP) such as bacterial lipopolycaccharide and peptidoglycan or fungal β-1,3 glucan binding to their respective pathogen recognition receptors. ProPO activation can also occur independently of PAMPs such as in the case of wounding and the presence of cells with altered apoptosis [55,57]. Activation of the proPO system results in the induction of a serine protease cascade but PO is activated by apolipophorin III and inhibited by lyzozyme and anionic peptide-2 in *G. mellonella* [58]. Consequently, the phenoloxidase activating system is initiated when prophenoloxidase-activating enzyme is converted from its inactive pro form (pro-ppA), to its active form (ppA). PpA can catalyse the proteolytic cleavage of prophenoloxidase (proP) to phenoloxidase (PO) (Figure 5). Active PO is involved in hydroxylation of monophenols. Hydoxylation is followed by the oxidation of phenols to form quinines. Finally, quinine polymerization is catalyzed by phenoloxidase-monophenyl-L-dopa to form melanin [59,60]. Excluding the final step in which melanin is produced, the proPO system displays similarities to the complement system of vertebrates. In both the complement system of mammals and the proPO system of insects, there is production of cytotoxic and opsonic components as summarized in Figure 5 [56]. Furthermore, there is some similarity between the sequences of insect proPO and the mammalian complement proteins C3 and C4 [61].

Although melanization is a vital component of the insect immune system, it must be tightly regulated by specific protease inhibitors to allow for accurate melanin deposition and to reduce the toxicity associated with melanin overproduction. One such example can be seen in *Drosophila* where the activation of the gene spn27A results in the production of a serpin [62]. This particular serpin functions by inhibiting the activation of the proPO system and therefore melanization [55]. There has been some debate on the role of melanization in *Drosphila*, however a comprehensive study by Binggeli *et al*. (2014) outlines the importance of PPO1 and PPO2 in dealing with Gram-positive bacteria and fungal infection [63].

In contrast, melanization in mammals plays quite a different role. Melanins (pheomelanin, eumelanin and neuromelanin) are produced by mammalian melanocytes in specific tissues including the brain, skin, hair and eyes. Melaninization plays a particularly important role in the skin where melanins protect the skin from UV by absorbing UVB rays and facilitates the production of Vitamin D3. Mammalian melanin production is a complex process that depends upon the levels of the antioxidant glutathione. At high glutathione levels, pheomelanin is produced while glutathione is not required for the production of eumelanin [64]. In insects injury or PAMP recognition initiates melanization, while in mammals melanization is initiated by either the hydroxylation of L-phenylalanine to L-tyrosine or direct hydroxylation of L-tyrosine to L-dihydroxyphenylalanine (L-DOPA), the precursor of both eumelanin and pheomelanin. After a number of oxidoreductions, the reaction intermediates dihydroxyindole and DHI carboxylic acid are produced before being polymerized to form eumelanin. Alternatively, pheomelanin is produced through the binding of dopaquinone to cysteine or gluthione producing cysteinyldopa and glutathionyldopa, respectively, before a series of reactions that eventually yield pheomelanin [65]. Overall, insect melanization displays more similarities to the mammalian complement system than to mammalian melanization due to insect melanization and complement playing an important role in microbial mediated innate immune responses, while mammalian melanization primarily plays a role in pigmentation and UV protection.

## Antimicrobial peptides in insects and mammals

**Figure 6.**
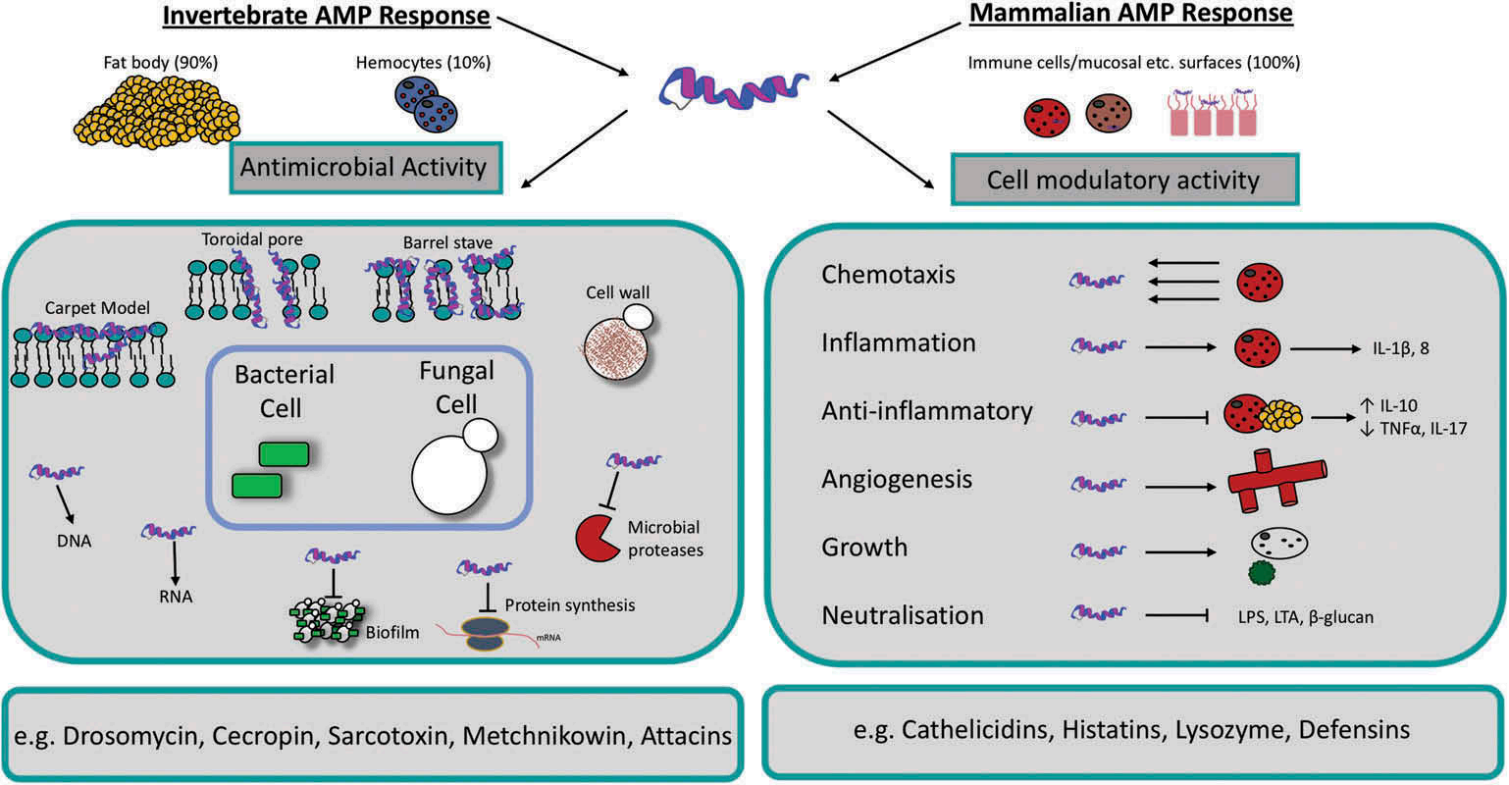
Schematic diagram of the mechanism of action and function of antimicrobial peptides in insects and mammals. Both insect and mammalian AMPs display direct microbicidal activity by initiating cell lysis at the cell surface or interfering with intracellular targets. Some AMPs possess anti-biofilm activity (e.g. LL-37), inhibit protein synthesis (e.g. apidaecin) or inhibit microbial proteases (e.g. Histatin-5). Some AMPs also possess pleotropic cell-modulatory activities such as angiogenesis, re-epithelization, chemotaxis, anti-inflammatory and growth effects depending on cell type.

Antimicrobial peptides (AMPs) are a group of widely expressed molecules that are produced as an early defence mechanism by multicellular organisms such as plants and animals. These peptides are produced in response to a broad spectrum of pathogens including bacteria, viruses, fungi and parasites but have also been found to target cancer cells [66,67]. Most of the identified AMPs share some common characteristics including a size of 12–50 amino acids, a net positive charge and an amphipathic structure. Furthermore, AMPs can be classified based on their secondary structure. These secondary peptide structures include α-helical, β-sheets, a mixture of α-helical and β-sheet structures or extended loop structures [68,69]. Depending on the particular AMP and its target pathogen, AMPs can target and kill pathogens using two distinct modes of action (Figure 6). Upon binding the microbial membrane, AMPs may induce cell lysis through disruption of the membrane. Alternatively, the peptide may induce pore formation through electrostatic interactions, allowing the AMP to target intracellular components of the pathogen such as DNA and RNA. Through binding intracellular proteins, the synthesis of DNA, RNA, proteins and the cell wall integrity may be altered resulting in cell death [70]. Additionally, AMPs have a chemotactic role in mammals which links the innate and adaptive immune response through recruiting and/or activating immune cells including T cells, dendritic cells and monocytes. For example human LL-37 induces chemotaxis of human neutrophils [71].

In mammals, AMPs are secreted from parts of the body that are subject to microbial infections including keratinocytes of the skin, the oral mucosa, gastrointestinal tract, lungs, eyes and the reproductive tract [72]. In insects, AMPs are predominantly synthesized in the fat body (equivalent to the mammalian liver) but also in small amounts by hemocytes before being secreted into the hemolymph [73–75]. As in mammals, the secretion of tissue-specific AMPs may also occur in the insect gut epithelia [66].

### Lysozyme

AMPs are extremely diverse with certain AMPs being found in particular animal species or groups while other AMPs may be conserved throughout different groups of species. One such example of an AMP conserved throughout different animal groups are lysozymes. Lysozymes are cationic proteins that target and kill Gram-positive bacteria through hydrolysing the peptidoglycan β-(1,4) glycosidic bonds in the bacterial cell wall [76]. Many lysozymes in insects and mammals also display chitinase activity which contributes to their antimicrobial function. Lysozymes are a diverse group and can be classified into six main sub-groups based on their activity, structure and source. These groups include chicken-type lysozyme (c-lysozyme) which is present in vertebrates and insects, goose-type lysozyme (g-lysozyme) which is mainly found in vertebrates and some molluscs, invertebrate-type lysozyme (i-lysozyme), plant lysozyme, bacterial lysozyme and phage lysozyme [77]. Lysozyme is found in the midgut and hemocytes of insects and the neutrophils, macrophages, monocytes, tears and saliva of mammals [78]. In mammals, lysozyme is expressed constitutively while expression is usually upregulated upon pathogen entry in insects [79]. Insect lysozymes are small proteins of approximately 14 kDa that display both functional and sequence similarities to mammalian lysozymes [80]. In particular, human lysozyme is a c-lysozyme consisting of a 130 amino acid polypeptide of approximately 14.7 kDa [81]. The *G. mellonella* transcriptome possesses four c-lysozyme and one i-lysozyme homologues, its protein is present in unstimulated larvae, is augmentable during infection, possesses antifungal activity, induces apoptosis in *Candida albicans* cells, acts in synergy with apolipophorin III and possesses immunomodulatory activity [82–86]. Upon comparative analysis, the c-lysozyme of *Musca domestica* was found to contain a 122-amino acid long polypeptide with a 38% sequence identity to human lysozyme [87]. Therefore, there are both structural and functional similarities found between insect and mammalian lysozymes. Although both mammalian and insect lysozymes have a role in immune defence, insect lysozymes may also have an additional digestive role in some insects due to its enzymatic properties in the midgut [87]. Pepsin mediated cleavage of lysozyme yields peptides which possess potent anti-inflammatory action on macrophages via interactions with TLR-4 [88]. This exemplifies the pleotropic activities of and cross talk between AMPs/proteins and the cellular immune response.

### Defensins

Defensins are an abundant group of AMPs found in insects and mammals and are characterized by a group of cysteine-rich cationic peptides that contain several disulfide bridges. Defensins are also small, ranging from 28–44 amino acids in size and display antimicrobial activity against a range of pathogens including bacteria (particularly Gram-positive bacteria), fungi and viruses [89,90]. Defensins function by targeting microbial cytoplasmic membranes and induce the formation of voltage-dependent ion channels. The formation of these ion channels alters the cell’s permeability therefore initiating the loss of cytoplasmic ions such as potassium. Ion loss ultimately results in microbial lysis [91,92]. In vertebrates, defensins are categorized into three groups based on their structure; α-defensins are present in neutrophils, macrophages and Paneth cells of mammals, β-defensins are present in mammalian neutrophils and epithelial cells while θ-defensins are believed to be exclusively present in primate neutrophils [93]. Interestingly, vertebrate β-defensins are more structurally similar to insect defensins than vertebrate α-defensins and θ-defensins, highlighting the conservation of defensins through evolution [66]. Human β-defensin 2 has a similar 3-D folded shape to insect defensin despite their differences in disulfide-bond distribution. Insect defensins, like mammalian defensins, have six cysteine residues involved in disulfide bond formation but are unique in that they have a protruding α-helical segment and are linked to the c-terminal of the β-sheet by two disulfide bridges [94–96]. In mammals, the β- defensin structure consists of cysteine residues that form three disulfide bonds from C1-C5, C2-C4 and C3-C6 [90].

Other antibacterial peptides are more specific and can only target either Gram-positive or Gram-negative bacteria, such as the invertebrate defensin isolated from *Formica rufa* (the red wood ant), which is active against Gram-positive bacteria [97]. Many AMPs display antifungal activity, some of which can also target bacteria, others only have activity against fungi [98]. Lebocin B, a proline-rich peptide from the insect *Manduca sexta*, shows antibacterial and antifungal properties.

### Proline-rich AMPs

Proline-rich AMPs can be isolated from mammals and insects and share common structural and functional characteristics. They possess unusually high amounts of proline residues, and often contain high levels of arginine producing a strong net positive charge and most target Gram-negative bacteria [99,100]. In bees such as *Apis mellifera* and the cicada killer bee *Sphecius speciousus*, apidaecins refer to a family of small, proline-rich peptides. These 18–20 residue peptides typically consist of two regions, a C-terminal conserved region that is responsible for the antimicrobial activity of the peptide, and an N-terminal variable region, which plays a role in extending the antibacterial spectrum of the peptide as seen in other AMPs. In this peptide, proline makes up 33% of the residues. Its amino nitrogen is cyclised with the side chain terminal carbon, restricting the structure of the peptide, such that it forms a polyproline helical type II conformation, which is an extended left handed helix with three residues per turn. The addition of multiple arginine residues in the conserved region creates a more positively charged molecule, possibly affecting its antibacterial activity [100]. In mammals, all short proline-rich AMPs belong to the cathelicidin family of AMPs [101]. The mammalian proline-rich peptides tend to be longer in contrast to those found in insects. PR-39 isolated from porcine small intestine and neutrophils, is a 39 residue long antibiotic peptide, composed of 49% proline and 24% arginine [102]. Similar to other Pro-AMPs, the presence of proline inhibits the peptide from adopting an alpha-helical conformation. Due to this it forms a polyproline helical type II, similar to apidaecins, but may undergo some slight conformational changes following binding to a lipid membrane [103]. Some studies suggest that PR-39 is the mammalian equivalent of apidaecins due to the similarity of their amino acid sequence and mode of action, however this view is still controversial [104]. Interestingly, proline-rich AMP Bac7(1–35) kills MRD *Pseudomonas aeruginosa* by disrupting their cell membranes, while it mode of action on *Escherichia coli* and *Salmonella enterica* serovar Typhimurium is primarily intracellular [105]. Furthermore, both insect oncocin and apidaecins have been demonstrated to bind to different regions of bacterial ribosomes, leading to inhibition of protein synthesis [106–108]. The fact these peptides act on multiple cellular targets makes bacterial resistance to their microbicidal activity difficult.

### Drosophila melanogaster AMPs

Although there is conservation of some AMPs such as defensins and lysozyme between insects and mammals there is far more variability between the two groups. At present over 290 AMPs have been identified in insects therefore it may be more informative to discuss insects AMPs in two species: *Drosophila melanogaster* and *Galleria mellonella*, (Table 1) [109]. In addition to defensins and lysozyme which predominantly target Gram-positive bacteria, *D. melanogaster* also express several AMPs that are not produced in mammals. For example, cecropins, drosocin, attacins, diptericin and maturated-pro-domain of attacin C (MPAC) are AMPs that target bacteria (mostly Gram-negative) while drosomycin and metchnikowin are AMPs that mostly target fungi in *Drosophila* [110].

Cecropins are amphipathic α-helical AMPs of 11 amino acids in length that have the ability to target and kill bacteria and filamentous fungi [94,111]. In insects, members of this family can be isolated from the hemolymph of moths and flies following bacterial infection [112–115]. These cecropins are 35–40 residues long, possess a C-terminal helical stretch and display a broad spectrum of activity against Gram-positive and Gram-negative bacteria, as well as some fungi [116]. Under aquatic solutions, these peptides typically adopt a random coil structure, but switch to an α-helical conformation in a hydrophobic environment. In Gram-negative bacteria, the hydrophobic C-terminal of cecropin interacts with the phospholipid membrane of the bacteria leading to membrane disruption and bacterial cell death [117]. It demonstrates antibacterial activity against multidrug resistant *A. baumanii* and *P. aeruginosa*, induces *C. albicans* apoptosis and recently has been shown to possess immunomodulary effects on macrophages [118,119]. There is evidence of cecropins in bovine adrenal glands and pig intestines, but they are more prominent in insects [80].

Metchnikowin is a proline-rich peptide consisting of 26 amino acid residues that has the ability to target both fungi and Gram-positive bacteria [120]. Metchnikowin specifically targets pathogenic fungi of the phylum Ascomycota such as *Fusarium* species. In the case of *Fusarium graminearum* strains, metchnikowin displays its fungicidal activity by targeting β (1,3)-glucanosyltransferase Gel1, an essential enzyme in cell wall biosynthesis. As a result, β(1,3)-glucan chain elongation is inhibited, disrupting cell wall synthesis and thus, initiating fungal cell death [121].

Attacins are glycine-rich AMPs of approximately 190 amino acids with a helical conformation and a random coil structure. In *Drosophila* species four genes, namely a*ttA, B, C* and *D* encode attacins [122]. Attacins kill Gram-negative bacteria by targeting lipopolysaccharide in the membrane resulting in an inhibition of protein synthesis. Attacins may also increase the permeability of the bacterial cell by creating ion channels in the membrane bilayer, initiating cell death [123].

### Galleria mellonella AMPs

*G. mellonella* larvae are now widely employed to study the virulence of a range of microbial pathogens [124–126] and to assess the immune responses of larvae to infection [127–130]. Their low cost, large size and ease of use, make them an ideal model system to overcome the disadvantages associated with mammalian testing and to generate comparable results in a short space of time [131]. *G. mellonella* produces at least 18 putative AMPs and their humoral response to a range of bacterial and fungal pathogens has been well documented in recent years with advances in transcriptomics and proteomic technologies [132,133].

Gallerimycin is a cationic 57 amino acid inducible cysteine-rich defensins peptide with anti-filamentous fungi activity against *Metarhizium anisopliae* and is inducible during bacterial infection [134–136]. Gallerimycin alone does not possess anti-bacterial activity but displays synergistic activity with cecropin A which expands the antimicrobial spectrum of gallerimycin by causing extensive non-lytic depolarization of *E. coli* membrane resulting in inhibition of growth [137].

Bioactivated galliomicin is a 43 amino acid AMP which contains 6 cysteine residues and exhibits anti-filamentous activity [135], is induced by *C. albicans* [138], physical stress [139] and extremes (4°C and 37°C) in temperature [140], indicating it is induced indiscriminately in times where infection may be likely. Other invertebrate α-helical AMPs include the moricins, isolated exclusively from the *Ledipoteran* insects. Moricins are 42-residues long α-helical peptides with 8 turns along the peptide. The N-terminal residues (5–22) are amphipathic and responsible for bacterial membrane permeability, while the C-terminal residues (23–36), are hydrophobic and needed for full antimicrobial activity [97]. Moricins are secreted as pro-peptides under the control of (NF-kB)/Rel and GATA transcription factors and are activated via proteolysis and increase the permeability of bacterial and fungal membranes. *G. mellonella* which has seven moricin-like peptides in its transcriptomics and these are highly active against yeasts and filamentous fungi [141]. Gloverins are glycine rich, heat stable antibacterial polypeptides believed to bind LPS and possibly components of the fungal cell wall. It was previously demonstrated that *E. coli* induces gloverin expression in *Bombyx mori* [142]. The abundance of both moricin-like peptides and gloverins are increased early during infection with *C. albicans* and *A. fumigatus* in *G. mellonella* larvae [128,143]. Larvae may also induce the expression/abundance of other humoral factors such as anionic peptide-1, Cecropin D-like peptide, hemolin, 27 kDa *G. mellonella* hemolymph protein, Hdd11 in order to curtail microbial growth before activation of the cellular immune response. With the recent release of the *G. mellonella* genome, there is much opportunity to study the role of individual humoral immune proteins/peptides during the infection process in larvae [144].

## Conclusion

The humoral component of the innate immune system of mammals shows many similarities to the insect immune response (Table 2). However there are also significant differences in processes and components of each response. The activation of NF-κB and its homologs in mammals and insects plays a very important role in immunity. In mammals, NF-κB is involved in production of cytokines and co-stimulatory molecules as well as cell survival and apoptosis signalling through the activation of Toll-like and TNF-α signaling pathways. In contrast, in *Drosophila* the NF-κB homologs (Dif, Dorsal and Relish) are activated by Toll and IMD pathways and are directly involved in innate humoral immune responses through initiating the transcription of AMPs. Blood/hemolymph clotting plays a vital role in both insect and mammalian immunity although clotting in insects is more important due to their open circulatory system. There are homologous clotting factors observed in both systems, particularly between insect transglutaminases and human clotting factor XIIIa. Specific clotting factors have also been identified such as hemolectin and Fondue in *Drosophila* and factors XII, XI, IX, VIII, X and V in humans. In mammals melanization plays a role in pigmentation and protection of the skin from solar radiation while in insects melanization plays a direct role in innate humoral immunity through activation of the proPO system by PAMPs. Defensins and lysozyme are two groups of AMPs found in both mammals and insects and mammalian c-lysozyme and β-defensins display structural and functional similarities to their insect counterparts. Furthermore, there are a variety of AMPs unique to particular animal groups including mammalian cathelicidins and insect metchnikowin, cecropin and attacins.

While there are many significant differences between the humoral component of the insect immune system and the innate immune system of mammals the similarities that are present are sufficient to allow the use of insects as models for studying microbial virulence. The increased use of insects as *in vivo* models for studying microbial virulence [12–14,124] or disease development [127–129,143,145] is to be welcomed. An enhanced understanding of the similarities and differences between the immune responses of insects and mammals will increase the attractiveness of using insects as *in vivo* models with a concomitant reduction in the use of mammals.

## Abbreviations

AMP: antimicrobial peptide
dFADD: Fas-associated death domain-containing protein
DHI: dehydrocholesterol
Gel: glucanosyltransferase
HNP: human neutrophil peptide
IL: Interleukin
IMD: immune deficiency
IMPI: insect metalloproteinase inhibitor
IRAK: interleukin-1 receptor-associated kinase
L-DOPA: L-dihydroxyphenylalanine
LRRs: leucine rich repeats
MPAC: Maturated-Pro-domain of Attacin C
MyD88: Myeloid differentiation primary response 88
NF-κβ: nuclear factor kappa B
PAMP: athogen-associated molecular pattern
PAR: Protease-activated receptor
PGRP: peptidoglycan recognition proteins
PO: phenoloxidase
ppA: prophenoloxidase-activating enzyme
TAB: TAK1 binding proteins
TAK: TGF- β –activating kinase
TF: tissue factor
TIR: Toll/interleukin-1 receptor
TLR: Toll-like receptor
TNFα: Tumor necrosis factor alpha
UV: ultraviolet
vWF: Von Willebrand factor.
